# An effective surgical educational system in the era of robotic surgery: “Double-Surgeon Technique” in robotic gastrectomy for minimally invasive surgery

**DOI:** 10.1007/s00423-024-03593-5

**Published:** 2024-12-28

**Authors:** Yoshihiko Kakiuchi, Shinji Kuroda, Yusuke Yoshida, Nobuhiko Kanaya, Hajime Kashima, Satoru Kikuchi, Shunsuke Kagawa, Toshiyoshi Fujiwara

**Affiliations:** Department of Gastroenterological Surgery, Okayama University Graduate School of Medicine, Dentistry and Pharmaceutical Sciences, 2-5-1 Shikata-Cho, Kita-Ku, Okayama City, Okayama, 700-8558 Japan

**Keywords:** Surgical education, Gastrectomy, Minimally invasive surgery, Robotic gastrectomy, Endoscopic surgical skill qualification system qualification

## Abstract

**Purpose:**

Gastric cancer (GC) remains a major malignancy. Robotic gastrectomy (RG) has gained popularity due to various advantages. Despite those advantages, many hospitals lack the necessary equipment for RG and are still performing laparoscopic gastrectomy (LG) due to its established minimal invasiveness and safety.

**Methods:**

This study assessed the effectiveness of the “Double-Surgeon Technique” (DST) for improving surgical education and proficiency with LG. The DST involves both a console-side surgeon and a patient-side surgeon working actively in RG, enhancing the skill acquisition needed for LG and potentially reducing surgical time. Assessment of this method was performed by surgical time, and cases were divided into three groups: first half (Phase 1: P1) and second half (P2) before the introduction of DST, and after the introduction of DST (P3).

**Results:**

Two surgical trainees were trained using the DST. The learning curve in both reached a plateau in P2, but descended again in P3. For one trainee, surgical time for P3 was significantly reduced compared to P1 (p = 0.001) and P2 (p = 0.0027) despite the intervals between laparoscopic distal gastrectomy as the main surgeon in P3 being significantly longer than in P2 (p = 0.0094). Other surgical results in both trainees did not differ significantly. Further, no difference in induction phase results of RG were evident between surgeons and trainees with or without DST experience.

**Conclusion:**

Surgical education using the DST could be effective in the current context of the need for RG and LG.

**Supplementary Information:**

The online version contains supplementary material available at 10.1007/s00423-024-03593-5.

## Introduction

Gastric cancer (GC) was the fifth most common malignancy and fourth leading cause of mortality worldwide in 2020 [1]. Surgical resection remains the standard strategy for GC, with or without adjuvant chemotherapy, in patients without distant metastases. The frequency of robotic gastrectomy (RG) has been steadily increasing every year due to the recognized advantages, such as precise movements, three-dimensional visualization, control of physiological tremor, and unrestricted range of motion. These advantages are well-established and supported by both short- and long-term evidence [2, 3]. However, not all hospitals are equipped with robots, so conventional open and laparoscopic gastrectomy (LG) will continue to be performed. LG is well known for being minimally invasive and safe as a curative procedure, is widely standardized in many hospitals, and its benefits are widely recognized [4]. As the number of GC cases declines, the proportion of RG is increasing, while LG are decreasing, In this context, it is essential to find methods to continue acquiring proficiency in LG techniques.

Various studies have explored the learning curve for LG. Previous research has suggested that a learning plateau in LG was reached between 10 and 20 cases [5, 6], with some reports indicating more than 40 cases [7–9]. In contrast, research into RG has suggested a learning curve plateau from 11 cases in one study [10], and less than 10 cases in others [11]. Shibasaki et al. concluded that RG reaches a plateau more quickly (in approximately 6–20 cases) than LG [12]. However, in many reports, RG was initiated after acquiring the Endoscopic Surgical Skill Qualification System (ESSQS) qualification, which certifies sufficient skills and experience in LG. This represents an obstacle to comparing learning curves between LG and RG. Although RG may become increasingly prevalent in the future, LG remains necessary at this time. In Japan, the standard approach to RD, typically involves the surgeon performing the entire procedure independently as a “solo surgery”, Key tasks such as dissection and clipping are carried out using robotic devices, with minimal involvement from assistants side. The primary role of the assistant is limited to exchanging robotic instruments. Because we considered that established surgical education methods for both RG and LG remain lacking, despite the need for training new trainees in both procedures, we analyzed a new surgical method that we advocate called the “Double-Surgeon Technique” (DST), which has an educational aspect. In DST, laparoscopic devices are used for tasks such as dissection and clipping, with assistants playing an active role in managing the surgical field. DST is an educational approach that enhances training efficiency by making beneficial use of opportunities for RG in addition to the limited opportunities for LG. This technique is currently uncommon in Japan but represents a compelling method that could potentially reshape the future of RG.

## Materials and methods

### Surgical concept and technique

The primary concept underlying the DST is to educate assistants as future surgeons. A byproduct of enhancing the skill level of assistants is a reduction in the operating time for RG. More precisely, DST aims to deepen the understanding of endoscopic surgical techniques and procedures through active surgical intervention by the patient-side surgeon. In certain institutions such as university hospitals, the education of surgeons is considered as important as improving surgical techniques.

**Fig. 1:**
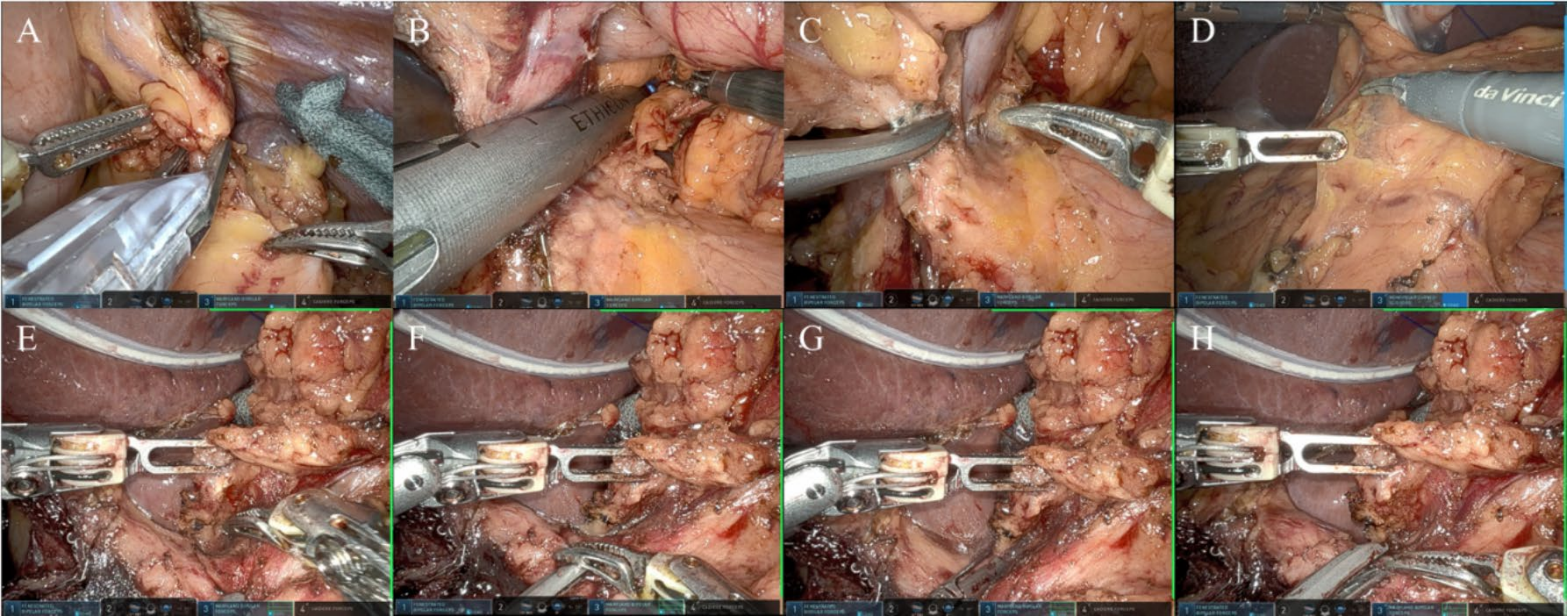
A series of images showing what the patient-side surgeon will do in DST**. A** The vascular clipping using clips **B** Stapling with an automatic suture device **C**) Vascular sealing and separation with an energy device **D**) Contribution to field development with grasping forceps **E**–**H**) Contribution to field development with suprapancreatic LND by grasping nerves with energy device and sealing and separating dissected tissue

The initial step of this process involves vascular clipping using clips and stapling with an automatic suture device during tissue dissection (Fig. 1A, B). This step is structured to allow mastery of the simplest techniques and can be reliably performed even by trainees with no surgical experience. On the other hand, unreliable clipping of blood vessels may lead to bleeding. In addition, this step helps trainees to comprehend the endpoint of lymph node dissection (LND). Stapling begins with the separation of the stomach, gradually proceeding to anastomosis. Since anastomosis is directly associated with postoperative complications, the console-side surgeon should develop tissue deployment initially, enabling trainees to concentrate on stapling and learn pitfalls more easily. The second step involves handling an energy device (Fig. 1C). This step is particularly crucial in LG. Determining the line of tissue dissection significantly influences LND. The console-side surgeon can guide in establishing the incision line, facilitating easy recognition of the incision by the trainee. As the trainee surgeon becomes more proficient, they can grasp the tissue and actively contribute to field development (Fig. 1D), thus reducing operation time. In addition, unanticipated bleeding may occur at unexpected sites due to the strength of the grasp and hand movements during dissection. Unlike LG, where both hands are in constant motion, the surgeon can focus solely on managing the energy device, concentrating on learning the appropriate grasping strength and controlling hand tremors. The final step is active intervention. This involves various elements, with a primary focus on deploying the field with assistance from the console-side surgeon, in addition to combining the first and second steps. Using an energy device facilitates repositioning of various organs without switching to forceps. Initially, tissue is grasped and deployed during the dissection of lymph node (LN) #4sb. Once the vessels are exposed, trainees perform clipping and utilize the energy device for dissection. By applying the techniques learned in the first and second steps, then mastering the delicate handling of tissue, trainees acquire the necessary knowledge for LND and refine their surgical techniques. The resulting advances in skill contribute to surgical efficiency, ultimately reducing the operation time. An even more advanced stage is intervention with suprapancreatic LND. During suprapancreatic LND, three robotic forceps and one assistant forceps enable utilization in the surgical field, similar to LG procedures. Specifically, when exposing the outermost layer of the common hepatic artery, the nerves are grasped by the energy device (Fig. 1E). Subsequently, the nerve grasp is transferred to the robotic arm (Fig. 1F), and LNs #12a and #8a are processed using the energy device (Fig. 1G). Following this, the energy device is again employed to grasp the nerves and deploy the field (Fig. 1H).

## Patients and data collection

This retrospective single-center study utilized data collected and analyzed at Okayama University Hospital, Okayama, Japan. The cohort comprised 60 patients who underwent laparoscopic distal gastrectomy (LDG) with D1 + LND and Billroth I reconstruction (B-I) for Stage IA GC performed by DST surgeon-1 (DSTs-1) from between January 2020 to September 2022 and by DSTs-2 from May 2022 to May 2024. DSTs-1 and DSTs-2 had performed 36 and 24 cases, respectively, and these 36 and 24 cases were divided into three groups; the first half, consisting of the first 11 and 9 cases (Phase 1: P1), and the second half, comprising the subsequent 11 and 8 cases (P2) before the introduction of DST, and the 14 and 7 cases after the introduction of DST (P3), respectively. As exclusion criteria, cases involving Roux-en Y reconstruction and D2 lymph node dissection were excluded during this period. DSTs-1 performed LDG with D2 LND in 3 patients and with D1 + LND and Roux-en-Y reconstruction (RY) in 6 patients. DSTs-2 also performed D1 + and RY in 6 patients (Supplementary Fig. 1).

In addition, RG proficiency during the RG induction phase was also examined according to DST experience. This comparison was conducted for robotic distal gastrectomy (RDG) with D1 + LND and either B-I or RY for Stage IA GC from January 2015 to June 2023. Three surgeons were enrolled: DSTs-1 and two surgeons through the conventional course (Cs-1, Cs-2), who did not have experience in DST. This comparison was limited to RDG and D1 + LND cases by the 15th cases after initiation of RG, and 5, 6, and 4 cases were performed by DSTs-1, Cs-1, and Cs-2, respectively. Before this period, DSTs lacked training experience in LDG, and no surgeons had experience in RDG (Supplementary Fig. 2).

Medical records of all patients were obtained from the hospital database. Patient factors (age, sex and body mass index [BMI]), surgical factors (time, bleeding, postoperative hospital days and complications by Clavien–Dindo grade [CD] [13]) were examined retrospectively, along with information about the surgeon.

## Statistical analysis

All statistical analyses were performed using JMP version 14.2 software (SAS Institute, Cary, NC, USA). Fisher’s exact test was used for categorical variables, and the Mann–Whitney *U* test and Steel–Dwass test were used for continuous variables. Probability values of *p* < 0.05 were considered significant.

## Results

### Patient clinicopathological characteristics and analysis of surgical time in LDG phase

**Fig. 2:**
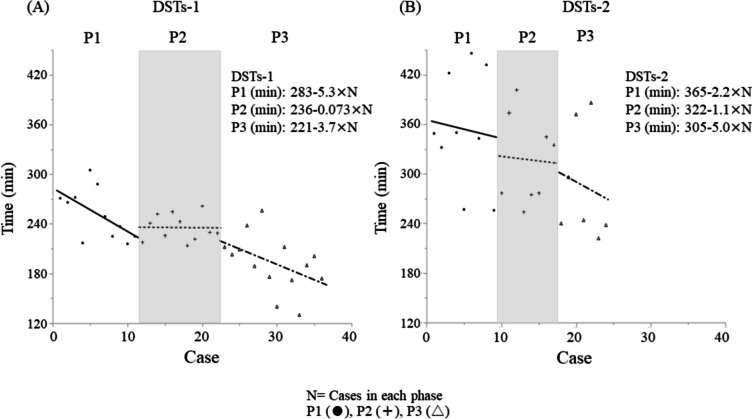
Surgical time plot for LDG and regression equation for surgical time for each phase (**A**: DSTs-1, **B**: DSTs-2)

Detailed information of surgeries by DSTs-1 and DSTs-2 is provided in Tables 1 and 2, respectively. Median ages of patients differed significantly between P2 and P3 (p = 0.042) for DSTs-1, but no significant differences in patient background were seen between DSTs-1 and DSTs-2. Surgical time in P3 for DSTs-1 showed a significant reduction compared to P1 (p = 0.001) and P2 (p = 0.0027). No significant differences in amount of intraoperative bleeding, postoperative hospital stay, or frequency of complications of CD Grade ≥ III were seen between DSTs-1 and DSTs-2. Mean interval between LDGs as the main surgeon was significantly longer in P3 than in P2 for DSTs-1 (p = 0.0094). For DSTs-2, although no significant differences were seen, the interval was approximately 1 month in all phases. Raw surgical times for LDG were plotted in chronological case order, and the regression equations for surgical time (in minutes) for each phase for DSTs-1 were P1: 283–5.3 × N, P2: 236–0.073 × N, and P3: 221–3.7 × N (N = cases in each phase) (Fig. 2A). For DSTs-2, these were P1: 365–2.2 × N, P2: 322–1.1 × N, and P3: 305–5.0 × N. Following the start of training, surgical time decreased (P1), then gradually leveled off (P2). After the introduction of DST (P3), surgical time again decreased (Fig. 2B).

**Table 1:** Patient characteristics and surgical outcomes in each phase for DSTs-1

		DSTs-1			P value		
**Phase (N)**		**P1 (11)**	**P2 (11)**	**P3 (14)**	**P1vsP2**	**P1vsP3**	**P2vsP3**
**Patient**	**Age, years (IQR)**	**67** **(63–76)**	**77** **(75–79)**	**68** **(60–73)**	**0.144**	**0.83**	**0.042**
	**Sex, M/F**	**6/5**	**9/2**	**6/8**	**0.36**	**0.7**	**0.099**
	**BMI, kg/m**^**2**^** (IQR)**	**22.4** **(20–25.2)**	**21.4** **(20.6–23.8)**	**21.8** **(20.4–24.7)**	**0.99**	**1**	**1**
**Surgery**	**Time, min (IQR)**	**249** **(225–272)**	**230** **(222–252)**	**196** **(174–212)**	**0.54**	**0.001**	**0.0027**
	**Bleeding, mL (IQR)**	**0** **(0–50)**	**0** **(0–10)**	**5** **(0–10)**	**0.99**	**1**	**1**
	**Postoperative hospital days, days (IQR)**	**11** **(10–15)**	**10** **(10–11)**	**10** **(9–13)**	**0.65**	**59**	**0.86**
	**Clavien-Dindo ≥ III, cases**	**2**	**0**	**1**	**1**	**1**	**1**
**Other**	**Interval between surgery, days (IQR)**	**17** **(7–42)**	**10** **(6–17)**	**45** **(14–66)**	**0.56**	**0.17**	**0.0094**

**Table 2:** Patient characteristics and surgical outcomes in each phase for DSTs-2

		DSTs-2			P value		
Phase (N)		P1 (9)	P2 (8)	P3 (7)	P1vsP2	P1vsP3	P2vsP3
**Patient**	**Age, years (IQR)**	**74** **(68–79)**	**79** **(77–82)**	**73** **(67–80)**	**0.25**	**0.55**	**1**
	**Sex, M/F**	**7/2**	**5/3**	**5/2**	**0.49**	**0.77**	**0.71**
	**BMI, kg/m**^**2**^** (IQR)**	**24.1** **(21.7–26.2)**	**24.1** **(21.5–27.7)**	**24.9** **(22.1–29.4)**	**0.86**	**0.61**	**0.73**
**Surgery**	**Time, min (IQR)**	**349** **(295–427)**	**306** **(278–367)**	**244** **(238–372)**	**0.57**	**0.17**	**0.44**
	**Bleeding, mL (IQR)**	**25** **(0–75)**	**28** **(5–125)**	**5** **(5–20)**	**0.85**	**0.8**	**1**
	**Postoperative hospital days, days (IQR)**	**12** **(10–17)**	**12** **(10–17)**	**10** **(10–13)**	**1**	**0.72**	**0.42**
	**Clavien-Dindo ≥ III, cases**	**2**	**2**	**2**	**0.89**	**0.77**	**0.88**
**Other**	**Interval between surgery, days (IQR)**	**37** **(14–71)**	**25** **(4–38)**	**28** **(21–35)**	**0.91**	**0.51**	**0.76**

This study focused on cases requiring D1 + lymph node dissection (cT1a/b N0M0) in which LDG was performed. While most cases were classified as pStage IA, the cohort also included two cases of pStage IB and Stage IIA, and one od pStage IIB. Regarding oncological outcomes, the median follow-up period for all cases was 2.7 and 1.0 years, respectively, with only one recurrence observed. This recurrence occurred as liver metastasis 6 months postoperatively in a case classified as pT1bN0M0 Stage IA following additional resection after ESD (Supplementary Fig. 3).

## Patient clinicopathological characteristics and analysis of surgical time in the RDG phase

**Fig. 3:**
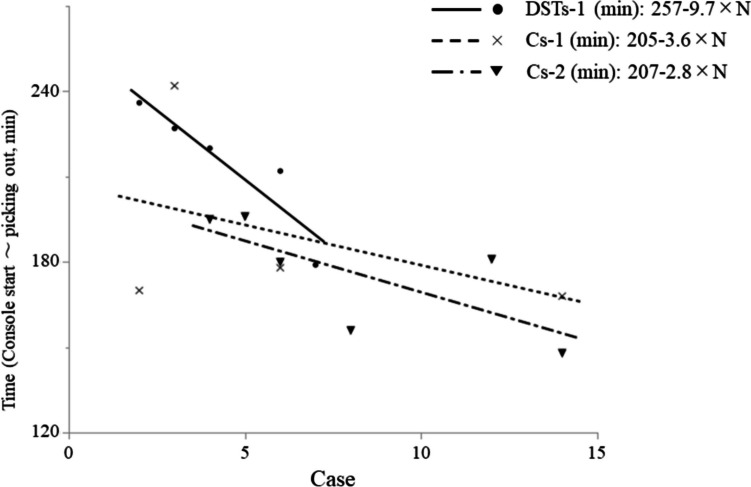
Surgical time plot for RDG during console start to picking up the stomach, and regression equation for surgical time of the three surgeons

Next, we investigated the advantage of the DST in the initial phase of RDG. This analysis compared DSTs-1, who had experience with the DST but no ESSQS qualification, with conventionally trained surgeons who had no experience with the DST, but had the ESSQS qualification. Detailed information is provided in Table 3. Median age and sex did not differ significantly between groups. Mean BMI of patients was 20.1, 25.5, and 25.4 kg/m^2^ for DSTs-1, Cs-1, and Cs-2, respectively, with a significant difference observed between DSTs-1 and Cs-1 (p = 0.037). No significant differences were observed in the time assessed from console start to picking up the stomach, bleeding, postoperative hospital stay, or frequency of complications of CD Grade ≥ III among the groups. Surgical times (in minutes) from console start to picking up the stomach were: DSTs-1: 257–9.7 × N; Cs-1: 205–3.6 × N; and Cs-2: 207–2.8 × N (N = cases by each surgeon on RDG) (Fig. 3).

**Table 3:** Patient characteristics and surgical outcomes for the three surgeons (DSTs-1, Cs-1, and Cs-2)

					P value		
Surgeon (N)		DSTs-1 (5)	Cs-1 (6)	Cs-2 (4)	DSTs-1 vs Cs-1	DSTs-1 vs Cs-2	Cs-1 vs Cs-2
Patient	Age, years (IQR)	68 (58–70)	67 (36–79)	77 (61–81)	1	0.36	0.73
	Sex, M/F	2/3	3/3	3/1	1	0.52	0.57
	BMI, kg/m2 (IQR)	20.1 (19.7–22.3)	25.5 (23.0–26.9)	25.4 (23.3–27.1)	0.037	0.052	1
Surgery	Time (Console start ~ picking up), min (IQR)	212 (187–232)	182 (170–193)	226 (170–255)	0.13	0.97	0.27
	Bleeding, mL (IQR)	40 (8–100)	100 (39–228)	10 (5–50)	0.43	0.36	0.051
	Postoperative hospital days, days (IQR)	12 (10–18)	14 (10–16)	9 (8–10)	1	0.085	0.056
	Clavien Dindo ≥ III, cases	0	1	0	1	NA	1

## Discussion

LG is widely standardized in many hospitals and its advantages are also widely recognized. The number of gastric surgeons who have obtained ESSQS qualification is gradually increasing, and the teaching system for LG is also improving. Actually, the utility of ESSQS qualifications has already been confirmed for LG, and outcomes can still be improved [14, 15]. However, the safety of RG has yet to be established and the educational method remains controversial. In general, education in this area is performed using a dual console system or an extra monitor on which important features can be indicated. These tools are considered superior tools for the RG education, but only one surgeon for one surgery can be trained using these tools. The RG surgeon must not only understand and operate the characteristics of the robot, but also understand the concept of GC surgery. A certain level of competence in operating a robot can be achieved using a simulation system[16]. However, preoperative preparation is important to understand the concept of GC surgery, and hands-on experience of the surgical process in the operating room is more important than textbook-based learning. In open and laparoscopic surgery, the role of the assistant surgeon is very important in the execution of surgery, but this role is declining in robot surgery because it includes elements of solo surgery. Therefore, improving the skills of assistant surgeons and maintaining their motivation may become problematic. DST can solve these problems by giving assistant surgeons roles in robotic surgery while maintaining the quality of surgical education. One advantage of the DST is the step-by-step nature of the educational system. The first step is to learn to operate the instruments necessary for LG. For grasping with forceps, the proper method to apply force and hold the forceps can be learned by actually holding the tissues in surgery. Clipping without trembling also helps prevent excessive bleeding. By ensuring the process of securely handling instruments in this manner, LG can be performed without complications due to instrument troubles. The next step is actual tissue dissection using the energy device. This stage involves applying the forceps grasp acquired in the first stage and deepening the understanding of surgery by actually performing the dissection. Active involvement in surgery is expected to increase the motivation of trainees and to improve their skills and understanding. The final step is the culmination of the skills and understanding acquired in previous steps. This step involves more difficult tasks such as deployment by grasping the nerves and dissection along the outermost layer. If this step can be performed without any problems, the operation time can be shortened as a result. DST is an effective system that partially delegates the task of RG to the patient-side surgeon, thereby assisting the surgeon to effectively acquire the necessary skills while still being an assistant.

We have shown in the present study that the educational system with the DST was successful for training with this concept. In particular, the learning curve plateaus once then descends again after experience with the DST, and this trend was observed in both trainees, representing a very impressive outcome. As numbers of RG increase and numbers of LG decrease, opportunities for trainee surgeons to perform procedures in both RG and LG remain limited. In the present study, although opportunities for trainees to perform procedures clearly decreased after the introduction of RG, the operating time for LDG was significantly reduced after introducing the DST. Another possibility is that the learning curve may reach a plateau similar to or even earlier than the general learning curve [5–9]. With the DST, after education with DST as the patient-side surgeon, the trainee can perform the surgery as the console-side surgeon. Another advantage of the DST is that the surgical process is almost the same for both LG and RG. RG can be thought of as simply a change from a laparoscopy to a robot device, as the console-side surgeon performs what the trainee has experienced on the assistant side. Another advantage of DST is that RG is usually a solo surgery, which means that field development and approach methods may change. The use of an energy device by the patient-side surgeon can also reduce the time expended in forceps exchanges. Another advantage of DST is that trainees who have completed training as the patient-side surgeon can begin training as the console-side surgeon, and a new trainee can begin training as the patient-side surgeon. This has advantages over the usual RG, which are being able to operate the robot and to understand the gastrectomy. In fact, in the present study, when RG was started after DST, the operative time was longer, but a significant time reduction became possible, reaching the same level as a regular RG surgeon within a few cases.

The present study may have some important implications for clinical practice, but also had several limitations. First, this study was not a randomized controlled trial, and instead retrospectively investigated a small cohort from a single institution. Second, the change to the DST was made midway after the start of regular LDG training. Therefore, there is a potential risk of bias regarding whether the observed effects are truly due to the educational impact of DST. However, given that the learning curve had already plateaued, it is reasonable to believe that DST may have had an influence.

In conclusion, education using DST may be effective in the current context of the need for both LG and RG. We hope that DST will aid in the development of better educational methods for trainees.

## Supplementary Information

Below is the link to the electronic supplementary material.

Supplementary figure 1 CONSORT diagram for the present study

High resolution image (TIF 78 KB)

Supplementary figure 2 An overview of DST program

High resolution image (TIF 122 KB)

Supplementary figure 3 Kaplan-Meier curve for relapse-free survival

High resolution image (TIF 82 KB)

Supplementary file4 (WMV 387740 KB)

## Data Availability

No datasets were generated or analysed during the current study.
