# Supplementary figures and images for: An effective surgical educational system in the era of robotic surgery: “Double-Surgeon Technique” in robotic gastrectomy for minimally invasive surgery
Source: Langenbecks Arch Surg. 2024 Dec 28;410(1):20. doi: 10.1007/s00423-024-03593-5 (PMC11682005; doi:10.1007/s00423-024-03593-5)

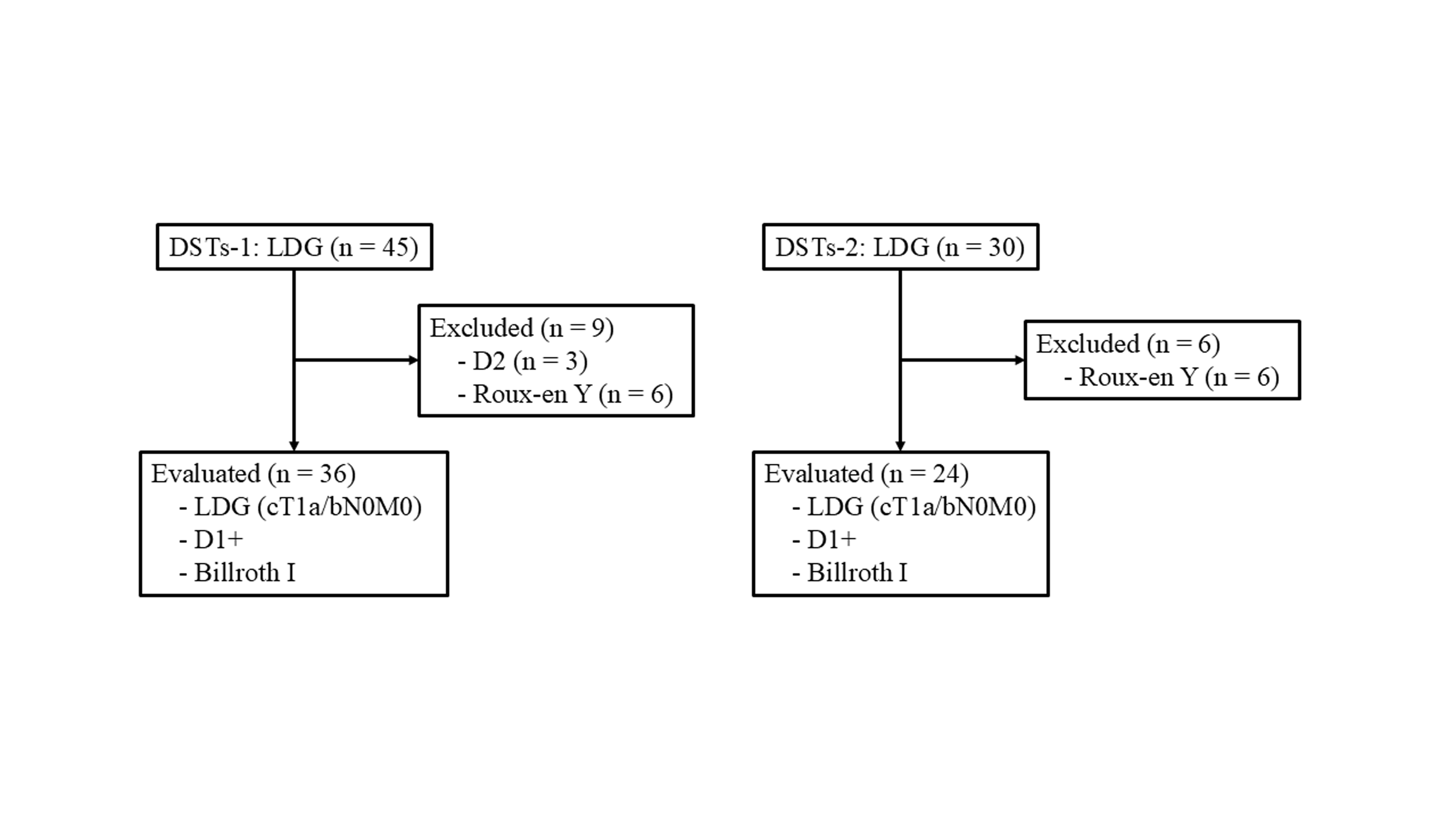

Supplement: Supplementary file 1 — Supplementary figure 1 CONSORT diagram for the present study [file 423_2024_3593_Fig4_ESM.png]

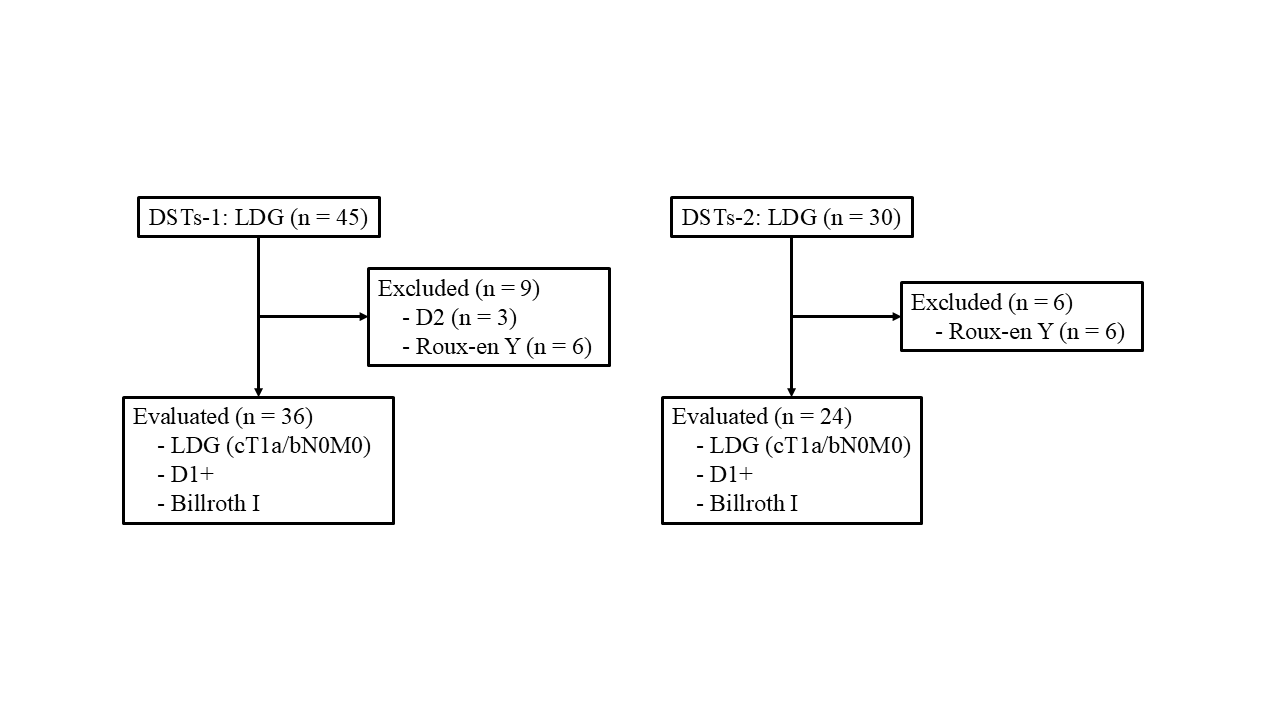

Supplement: Supplementary file 2 — High resolution image (TIF 78 KB) [file 423_2024_3593_MOESM1_ESM.tif]

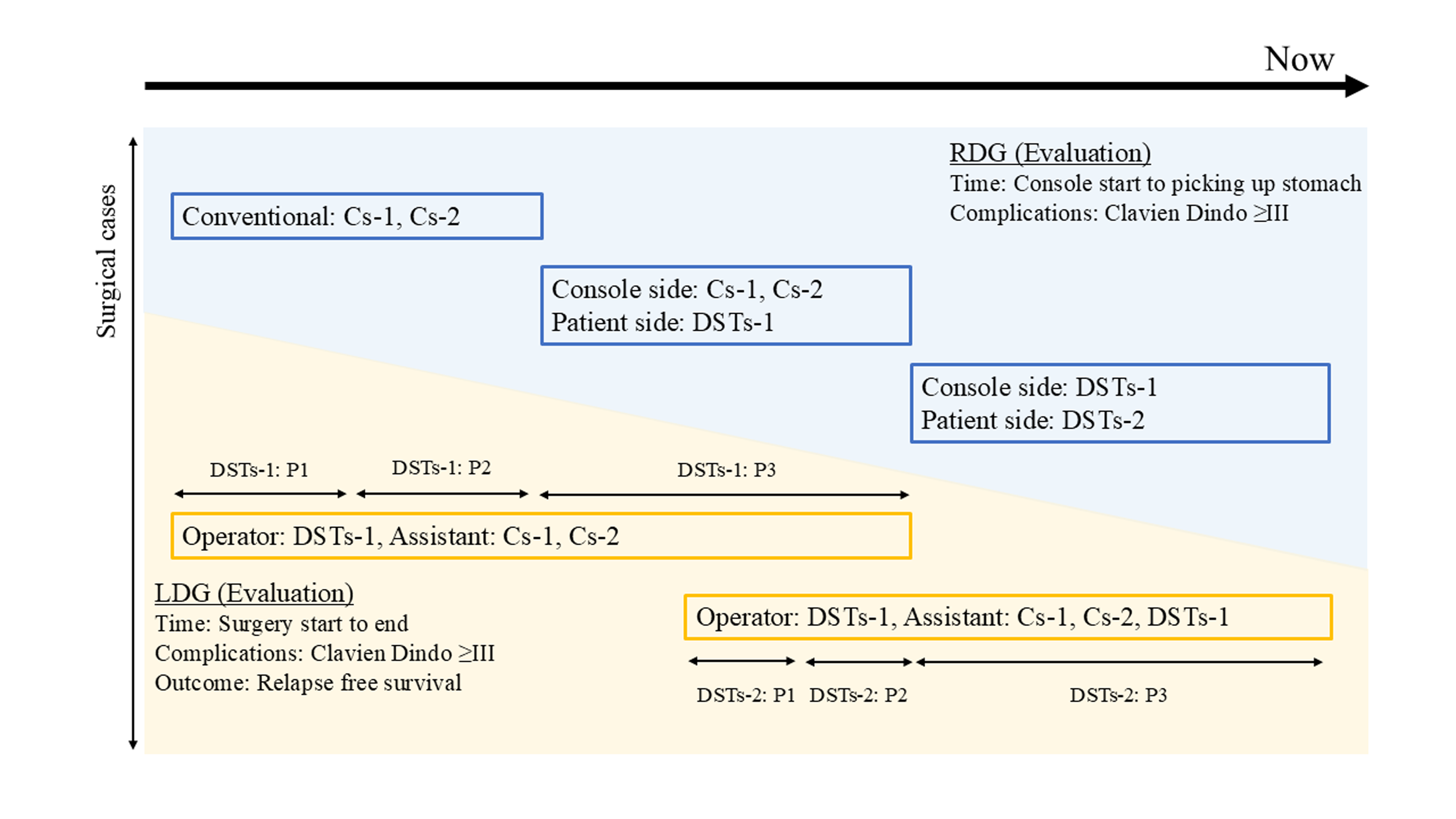

Supplement: Supplementary file 3 — Supplementary figure 2 An overview of DST program [file 423_2024_3593_Fig5_ESM.png]

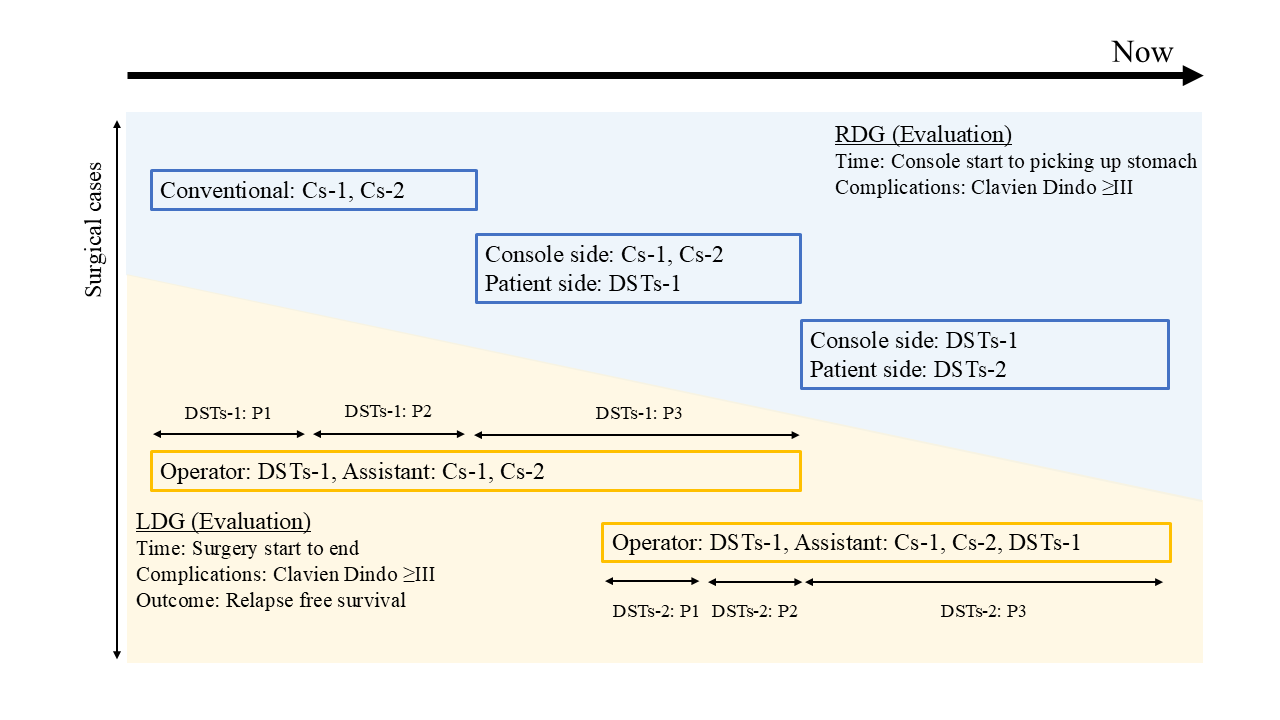

Supplement: Supplementary file 4 — High resolution image (TIF 122 KB) [file 423_2024_3593_MOESM2_ESM.tif]

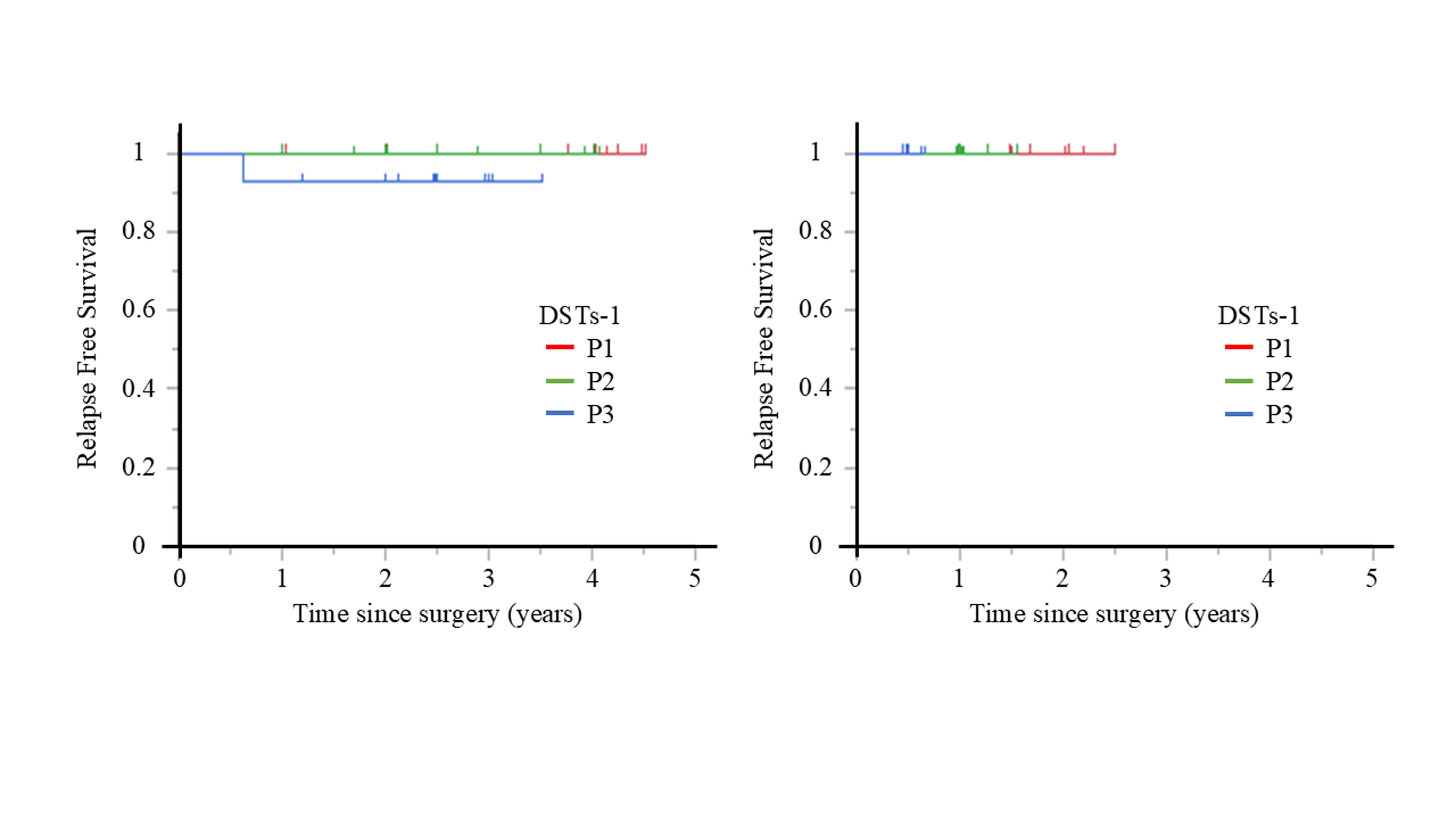

Supplement: Supplementary file 5 — Supplementary figure 3 Kaplan-Meier curve for relapse-free survival [file 423_2024_3593_Fig6_ESM.png]

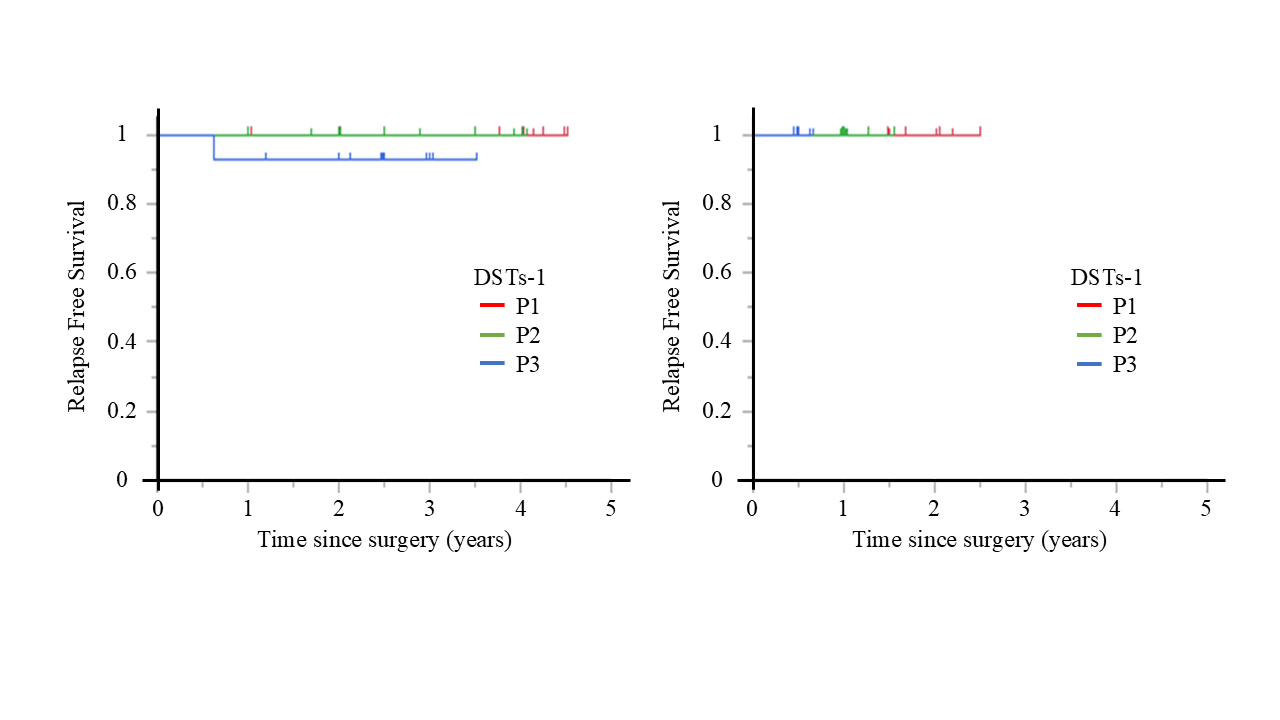

Supplement: Supplementary file 6 — High resolution image (TIF 82 KB) [file 423_2024_3593_MOESM3_ESM.tif]
